# Comparative Investigation of Phenomenological Modeling for Hysteresis Responses of Magnetorheological Elastomer Devices

**DOI:** 10.3390/ijms20133216

**Published:** 2019-06-30

**Authors:** Yang Yu, Jianchun Li, Yancheng Li, Shaoqi Li, Huan Li, Weiqiang Wang

**Affiliations:** Centre for Built Infrastructure Research, School of Civil and Environmental Engineering, University of Technology Sydney, Ultimo, NSW 2007, Australia

**Keywords:** magnetorheological elastomer device, phenomenological model, model identification, hysteresis

## Abstract

Magnetorheological elastomer (MRE) is a type of magnetic soft material consisting of ferromagnetic particles embedded in a polymeric matrix. MRE-based devices have characteristics of adjustable stiffness and damping properties, and highly nonlinear and hysteretic force–displacement responses that are dependent on external excitations and applied magnetic fields. To effectively implement the devices in mitigating the hazard vibrations of structures, numerically traceable and computationally efficient models should be firstly developed to accurately present the unique behaviors of MREs, including the typical Payne effect and strain stiffening of rubbers etc. In this study, the up-to-date phenomenological models for describing hysteresis response of MRE devices are experimentally investigated. A prototype of MRE isolator is dynamically tested using a shaking table in the laboratory, and the tests are conducted based on displacement control using harmonic inputs with various loading frequencies, amplitudes and applied current levels. Then, the test results are used to identify the parameters of different phenomenological models for model performance evaluation. The procedure of model identification can be considered as solving a global minimization optimization problem, in which the fitness function is the root mean square error between the experimental data and the model prediction. The genetic algorithm (GA) is employed to solve the optimization problem for optimal model parameters due to its advantages of easy coding and fast convergence. Finally, several evaluation indices are adopted to compare the performances of different models, and the result shows that the improved LuGre friction model outperforms other models and has optimal accuracy in predicting the hysteresis response of the MRE device.

## 1. Introduction

As a novel type of smart materials, magnetorheological elastomer (MRE) has received remarkable recognition in engineering applications. With micro-sized magnetic particles dispersed in the elastomeric matrix and formed as chain-like structures during curing, MRE behaves as normal rubber-like materials when no magnetic field is applied and exhibits instantaneous and reversible changes in mechanical properties upon application of a magnetic field [1,2,3,4]. The most noticeable change in material property of MRE under a magnetic field is its shear modulus [5,6], which achieves a relative percentage increase from about 50% to 1300% at saturation, for stiffer and softer carrier, respectively [7,8]. Additionally, MRE also expresses variable damping [9,10,11]. These unique features indicate that MRE can be adopted for designing controllable devices such as adaptively tuned vibration absorbers [12,13,14,15,16,17] and isolators [18,19,20,21,22,23,24], stiffness tunable mounts and suspensions [25], and variable impedance surfaces [26].

Successful implementations of MRE have been reported in both mechanical and civil engineering applications for vibration mitigation. Ginder et al. proposed the first tuned vibration absorber (TVA) and suggested that the field dependency of modulus was still available when the applied frequency well exceeded 1 kHz [25]. Since then, studies on applications of MRE have been widely conducted. Zhang and Li constructed and tested an adaptively tuned vibration absorber which can alter its natural frequency from 35 to 90 Hz when the applied current increased from 0 to 3 A [17]. Utilizing the feature of controllable stiffness and damping of MRE, Behrooz et al. designed a variable stiffness and damping isolator to explore its feasibility as an adaptive base isolator in civil infrastructures [27]. Addressing the issue of vertical loading capacity, Li et al. proposed the first adaptive laminated isolator, achieving a 37% increase in effective stiffness when powered by a 5 A current [22]. Bastola and Li studied the influence of the magnetic field and vertical loading to an MRE-based isolator by a simple setup with a 730-foldincrease of its original stiffness [23].

When MRE devices are employed for structural vibration suppression, the corresponding control systems should be developed to allow semi-active control of the devices for protecting the structures against external hazard vibrations. Although these devices have promising prospects in sufficient vibration mitigation, the main challenge for their engineering application is to model their highly nonlinear and hysteretic stress–strain/force–displacement behavior. As commonly known, in order for the semi-active control system to be designed and implemented for structural vibration control, physically, numerically traceable, and computationally efficient models that can portray this unique behavior well are required. Generally, the developed models for MRE device should have a simple structure and avoid the piecewise function and differential equations. Besides, the number of model parameters should be as few as possible so that the corresponding real-time controller is easy to implement in practice. To meet this requirement, different researchers have proposed various modeling methods for the dynamic behaviors of the MRE materials and devices, which are generally categorized as parametric and nonparametric modeling. For instance, the Kelvin–Voigt model and four-parameter model were employed by Zhao et al. and Li et al. to predict the hysteretic force–displacement responses of the MRE devices under shear loading [21,28]. In [29], the phenomenological model constituted by a standard linear solid model, a stiffness-adjustable spring, and an interfacial slip component were designed to depict the rheological characteristic of the MRE material. A hyperbolic sine component was introduced in the Kelvin–Voigt model to illustrate nonlinear strain stiffening phenomenon when the MRE is supplied with increasing magnetic field [30]. Furthermore, complex hysteresis models with highly nonlinear differential equations, such as the Bouc–Wen model [31,32], Dahl model [26], improved LuGre friction model [33], and the strain stiffening model [22], were developed to characterize the MR effect on device behavior. On the other hand, with the rapid development of artificial intelligence (AI) techniques, nonparametric methods have become more and more popular to model MRE devices via self-adaptive learning algorithms. A standard three-layer perception network was proposed by Koo et al. to demonstrate the dynamic responses of the MRE material under compressive loading [34]. However, such a network is static and has low modeling accuracy because of the shortage of internal memory. To fix this drawback, Fu et al. proposed a nonlinear autoregressive network with ectogenous inputs to simulate the nonlinear relationship between the input vector of velocity, displacement, applied current, and output shear force of the MRE isolator [35]. Moreover, fuzzy neural network and extreme learning machine were reported as novel nonparametric methods to simulate the dynamic behavior of the MRE device [36,37]. Although the nonparametric models have higher modeling accuracies than parametric models, they suffer from the problem that the model parameters (e.g., connection weights and bias in network) do not have any physical meaning and cannot directly be related to the MR effect of the device. Accordingly, a model with parameters that can effectively explain the MR phenomenon is urgently needed to design the semiactive controller for better implementation of the MRE devices in vibration control.

This paper aims to characterize the nonlinear dynamic behavior of MRE-based devices via numerical modeling, which is beneficial to the design of real-time controllers in these semiactive devices’ engineering applications. First of all, the up-to-date phenomenological models for the MRE material and devices are summarized, including the Kelvin–Voigt model, four-parameter model, rheological model, Bouc–Wen (BW) model, improved BW model, Dahl model, improved LuGre friction model, strain stiffening model, and the hyperbolic hysteresis model. Detailed descriptions of these models are provided in the following section. Then, the parameters of different models are identified using experimental data, which were captured for the dynamic tests of a prototype MRE isolator based on harmonic excitations from a shaking table in the laboratory. Model parameter identification is a global optimization procedure, in which the genetic algorithm (GA) was employed to search for optimal model parameters. Finally, the model performances for predicting dynamic behavior of the MRE device are comprehensively examined based on force–displacement hysteresis loops, mechanical properties such as effective stiffness and equivalent damping, and statistical evaluation indices.

## 2. Overview of Phenomenological Models for MRE Materials and Devices

### 2.1. Kelvin–Voigt Model

**Figure 1:**
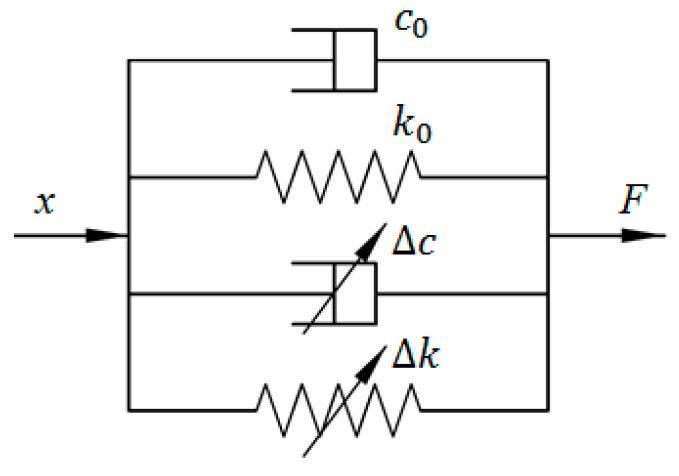
Kelvin–Voigt model.

Zhao et al. adopted the classical Kelvin–Voigt model to portray the viscoelastic characteristics of MRE-based bearings [21], the schematic of which is shown in Figure 1. In this model, the springs are connected to the dashpots in parallel to illustrate the nonlinear force–displacement relationship, where *k*_0_ and *c*_0_ respectively denote the zero-magnetic stiffness and damping, while ∆*k* and ∆*c* respectively represent magnetic stiffness and damping. The relationship between output force *F* and displacement *x* can be expressed using the following equation:

(1) $$F=\left(\Delta c+c_{0}\right)\dot{x}+\left(\Delta k+k_{0}\right)x=c_{ef}\dot{x}+k_{ef}x$$

where *k_ef_* and *c_ef_* denote the equivalent stiffness and equivalent damping, respectively; *x* and $\dot{x}$ denote the displacement and velocity of the device.

Testing results in [21] show that the equivalent stiffness and damping are the functions of applied current *I* and excitation amplitude *A*. Therefore, the relationship between equivalent stiffness and current and amplitude can be expressed as follows:

(2) $$k_{ef}=\alpha A^{2}+\beta A+\gamma I^{2}+\delta I+\tau$$

where *α*, *β*, *γ*, *δ*, and *τ* are parameters to be identified. Similarly, the relationship between equivalent damping and current and amplitude is shown in Equation (3):

(3) $$c_{ef}=aA^{3}+bA^{2}+cA+dI^{2}+eI+g$$

where *a*, *b*, *c*, *d*, *e*, and *g* are parameters to be identified. Substitute Equations (2) and (3) into Equation (1) and the mathematical expression of the output force *F* can be rewritten as Equation (4).

(4) $$F=\left(aA^{3}+bA^{2}+cA+dI^{2}+eI+g\right)\dot{x}+\left(\alpha A^{2}+\beta A+\gamma I^{2}+\delta I+\tau \right)x$$

### 2.2. Four-Parameter Viscoelastic Model

**Figure 2:**
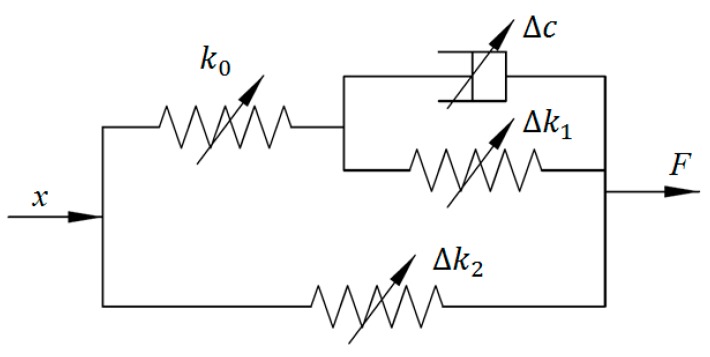
Four-parameter viscoelastic model.

Li et al. proposed a novel viscoelastic model with four tuned parameters to characterize the mechanical behavior of MRE devices [28], the schematic of which is shown in Figure 2. It is noticeable that this model is an extension of standard viscoelastic model, where a linear spring is added in parallel with the three-parameter model to illustrate the field-dependence of the MRE device. In the figure, the damping capacity of the device is represented by a standard viscoelastic model consisting of *k*_1_, *c*_2_, and *k*_2_, while the field-dependent stiffness is expressed by *k_b_*. *x* and *F* denote the displacement and force of the device. Hence, the relationship between force *F* and displacement *x* can be expressed as follows:

(5) $$\left(1+\frac{k_{2}}{k_{1}}\right)F+\frac{c_{2}}{k_{1}}\dot{F}=\left(k_{b}+\frac{k_{b}k_{2}}{k_{1}}+k_{2}\right)x+c_{2}\left(1+\frac{k_{b}}{k_{1}}\right)\dot{x}$$

Since the MRE has the characteristic of linear viscoelasticity, the force *F* can be represented by Equations (6) and (7).

(6) $$F=Kx=\left(K_{1}+iK_{2}\right)x$$

(7) $$\dot{F}=K\dot{x}$$

where *K* denotes the complex stiffness, and *K*_1_ and *K*_2_ are corresponding real part and imaginary part, respectively. Linear viscoelastic theory can be used to obtain the expressions of *K*_1_ and *K*_2_, shown as follows:

(8) $$K_{1}=\frac{\left(k_{1}k_{b}+k_{2}k_{b}+k_{1}k_{2}\right)\left[{\left(k_{1}+k_{2}\right)}^{2}+c_{2}^{2}\omega^{2}\right]+c_{2}^{2}\omega^{2}k_{1}^{2}}{\left(k_{1}+k_{2}\right)\left[{\left(k_{1}+k_{2}\right)}^{2}+c_{2}^{2}\omega^{2}\right]}$$

(9) $$K_{2}=\frac{c_{2}\omega k_{1}^{2}}{{\left(k_{1}+k_{2}\right)}^{2}+c_{2}^{2}\omega^{2}}$$

where *ω* denotes the excitation frequency. Assume there is a sinusoidal input displacement *x* with the following expression:

(10) $$x=x_{0}\sin\left(\omega t\right)$$

where *x*_0_ denotes the excitation amplitude. The stable force response of the MRE device can be expressed as Equation (11):

(11) $$F\left(t\right)=x_{0}\sqrt{K_{1}^{2}+K_{2}^{2}}\sin\left(\omega t+\varphi \right)$$

where *φ* denotes the phase angle shift between input displacement and output force, and its expression is given by:

(12) $$\varphi =\tan^{-1}\left(\frac{K_{2}}{K_{1}}\right)$$

### 2.3. Rheological Model

**Figure 3:**
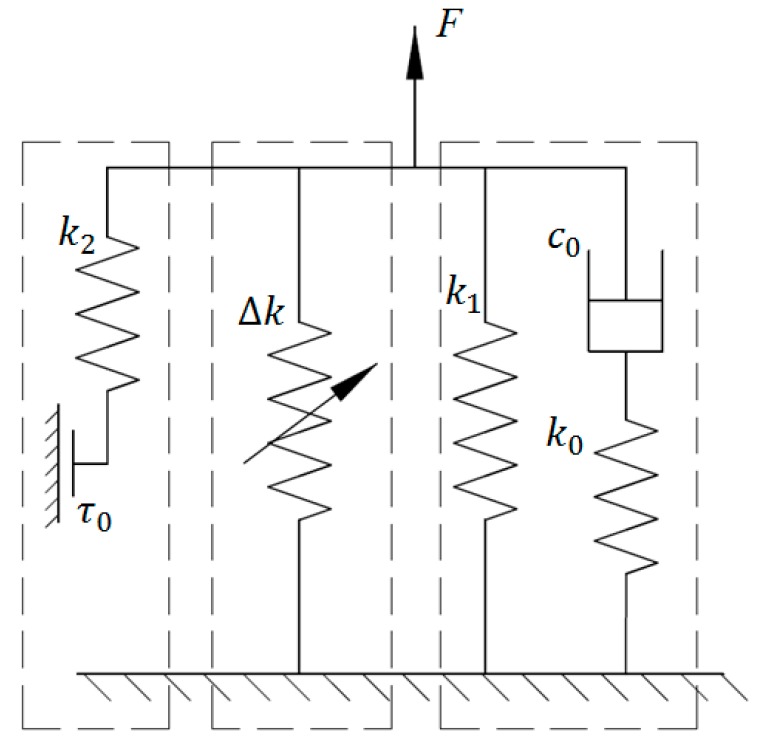
Rheological model.

To consider the effect of the interactions between polymer matrix and embedded particles on mechanical properties, Chen and Jerrams employed the rheological theory to develop a phenomenological model for MRE materials [29]. This model can be divided into three parts to respectively depict linear viscoelastic behavior, field-dependent mechanical properties, and interface slip between the particles and matrix, which is shown in Figure 3.

Since the MRE has the viscoelastic material of rubber, its mechanical properties without magnetic effect can be formulated using a three-parameter solid model, as shown in the right part of Figure 3. When external load is applied to this element, the resultant shear strain is very small and has a linear relationship with shear stress. Similarly, the shear stress should be proportional to the shear strain rate. The relevant expressions are provided as follows:

(13) $$\tau_{1}=G_{1}\gamma$$

(14) $$\tau_{2}=G_{2}\gamma_{2}$$

(15) $$\tau_{2}=\eta \dot{\gamma_{1}}$$

(16) $$\gamma =\gamma_{1}+\gamma_{2}$$

where *G*_1_ and *G*_2_ denote the shear modulus of two springs; *γ_i_* (*i* = 1 or 2) and *τ_i_* (*i* = 1 or 2) denote the corresponding shear strain and shear stress, respectively; *η* denotes viscoelastic property of the dashpot.

On the other hand, external magnetic fields can magnetize the ferromagnetic particles in MREs and contribute to the field-dependent modulus due to interaction force among particles. To explain this magnetic field-induced phenomenon, a changeable stiffness element is added in the model, which is the middle part in Figure 3. The shear stress caused by magnetism can be obtained by calculating the derivative of dipole energy density regarding strain, shown in Equation (17):

(17) $$\tau_{3}=\frac{\partial U}{\partial \gamma }$$

where *τ*_3_ denotes the shear stress induced by magnetic fields; *U* denotes the dipole energy density and can be represented by:

(18) $$U=\frac{\phi^{2}\left(\gamma^{2}-2\right)J_{p}^{2}}{8\pi \mu_{1}\mu_{0}\pi {\left(1+\gamma^{2}\right)}^{\frac{5}{2}}}$$

where *μ*_1_ denotes relative medium permeability; *ϕ* denotes the particle volume proportion in MRE; *J_p_* denotes the moment amplitude of dipole per unit particle volume.

Furthermore, Li et al. pointed out that interfacial adhesion between particles embedded and neighboring matrix can also affect the mechanical properties of MRE [38]. It is indicated that with the increase of interfacial bonding strength, the damping property is reduced while the shear modulus of the MRE is inversely increased. In [29], a Coulomb friction element together with a linear spring is introduced to model interfacial interaction, which is the first branch of the left in Figure 3. When external load arrives at the critical point, the spring will be kept unchanged and the Coulomb friction element starts to slide. The corresponding mathematical relationship can be expressed as follows:

(19) $$\dot{\tau_{4}}=G_{4}\dot{\gamma \;},\;\|\tau_{4}\|<\tau_{c}$$

(20) $$\tau_{4}=\mathrm{sgn}\left(\dot{\gamma }\right)\tau_{c},\;\|\tau_{4}\|\geq \tau_{c}$$

where *τ*_4_ denotes the stress caused by spring and Coulomb friction elements; *G*_4_ denotes the spring shear modulus; ‘sgn’ denotes the sign function; *τ_c_* denotes the critical stress. Combining the results of all the components together, we can get the constitutive relationship between the stress and strain as follows:

(21) $$\tau =\tau_{1}+\tau_{2}+\tau_{3}+\tau_{4}$$

### 2.4. Bouc–Wen Model

**Figure 4:**
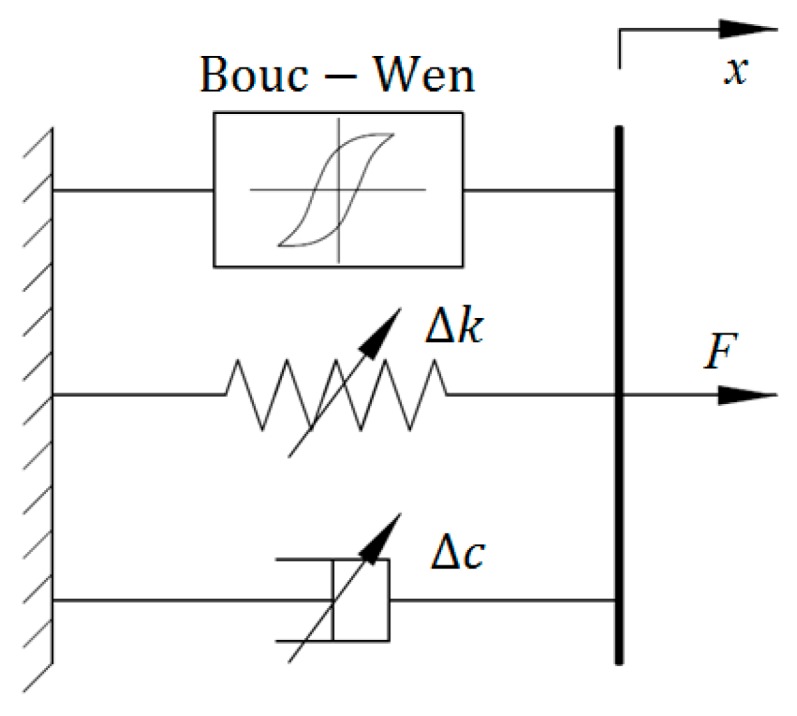
Bouc–Wen model.

To characterize the strain stiffening phenomenon and nonlinear force–velocity relationship of MRE device, Yang et al. proposed a Bouc–Wen (BW)-based phenomenological model in which the Voigt and BW components are connected in parallel to represent solid material behavior and hysteresis, respectively [31]. The schematic of the Bouc–Wen model for the MRE device is shown in Figure 4. The shear force of this nonlinear system is formulated as follows:

(22) $$F=\alpha k_{0}x+\left(1-\alpha \right)k_{0}z+c_{0}\dot{x}$$

where *x* and $\dot{x}$ denote the displacement and velocity of the MRE device, respectively; *k*_0_ and *c*_0_ denote the stiffness and viscous coefficients of the system, respectively; *α* represents the scaling parameter in the range of [0, 1]; *z* is an intermediate variable indicating the device displacement’s time history. The mathematical expression of *z* is given in Equation (23).

(23) $$\dot{z}=A\dot{x}-\beta \left|\dot{x}\right|{\left|z\right|}^{n-1}z-\delta \dot{x}{\left|z\right|}^{n}$$

where $\dot{z}$ denotes the derivative of *z* in time domain; *A*, *β*, *n* and *δ* are nondimensional parameters that regulate the size and shape of the hysteretic loop. Among all the parameters in the Bouc–Wen model for MRE device, *k*_0_, *c*_0_, *α*, and *A* are field-dependent. Their relationships with applied current *I* can be expressed by the first-order polynomial functions, which are shown in Equations (24)–(27).

(24) $$k_{0}=k_{0a}+k_{0b}I$$

(25) $$c_{0}=c_{0a}+c_{0b}I$$

(26) $$\alpha =\alpha_{a}+\alpha_{b}I$$

(27) $$A=A_{a}+A_{b}I$$

### 2.5. Improved Bouc–Wen Model

**Figure 5:**
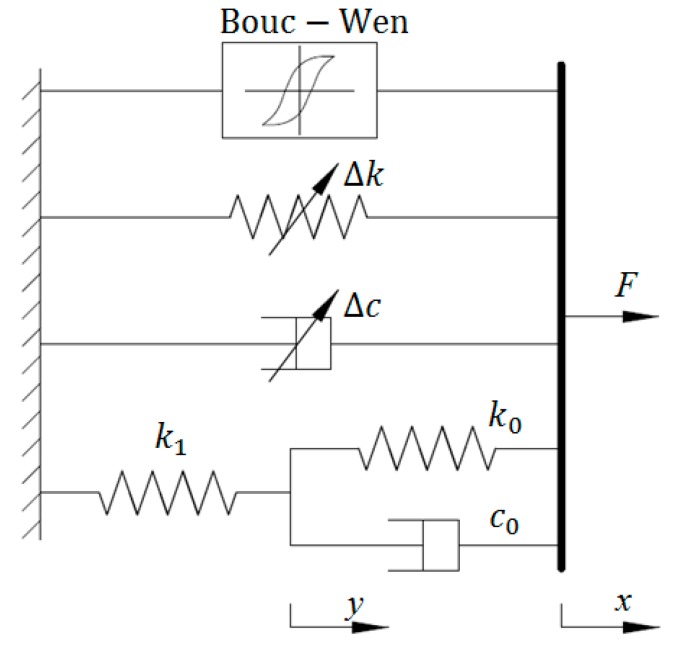
Improved BW model.

Based on the classical BW model, Behrooz et al. developed an improved BW model to describe the damping, stiffness, and hysteresis behaviors of a MRE-based variable stiffness and damping isolator (VSDI) [32], the schematic of which is shown Figure 5. It is clearly seen that this model adopts a field-dependent dashpot, a field-dependent spring element, and a field-dependent BW component to signify the changes of damping, stiffness, and hysteresis of the VSDI with regard to the increase of the applied current. The generated force of the model can be expressed as follows:

(28) $$F=c_{2}\left(\dot{x}-\dot{y}\right)+k_{2}\left(x-y\right)+c_{m}\dot{x}+k_{m}x+\alpha z$$

where *α*, *k*_2_, and *c*_2_ are constant; *k_m_* and *c_m_* are field-dependent stiffness and damping coefficients. *z* is the intermediate variable and its expression is same as that in the classical BW model (Equation (22)). *y* is another intermediate variable which can be obtained according to the force equilibration:

(29) $$c_{2}\left(\dot{x}-\dot{y}\right)+k_{2}\left(x-y\right)=k_{1}y$$

where *k*_1_ denotes a stiffness constant parameter. Similar to the classical BW model, this improved BW model considers the linear relationships between field dependent parameters and applied current *I*, which are shown in Equations (30)–(32).

(30) $$k_{m}=k_{m0}+k_{m1}I$$

(31) $$c_{m}=c_{m0}+c_{m1}I$$

(32) $$\alpha_{m}=\alpha_{m0}+\alpha_{m1}I$$

### 2.6. Dahl Model

**Figure 6:**
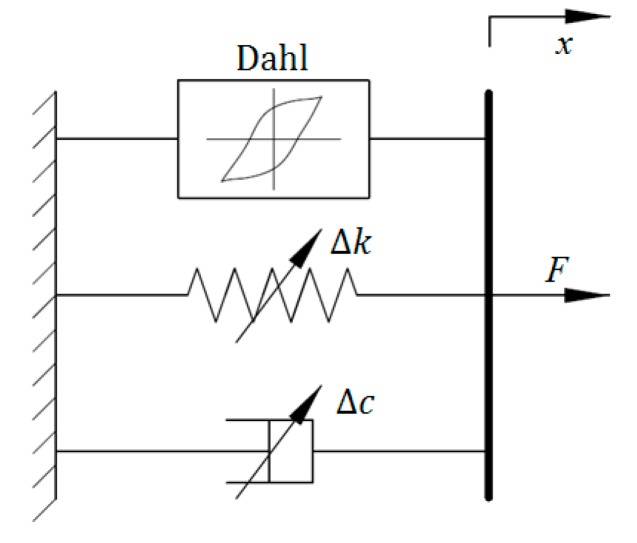
Dahl model.

The Dahl model was initially designed to simulate the control system with the feature of friction. It can be used to model nonlinear force–displacement loops via the differential equation. In [26], an effective and simple improved Dahl model was developed to characterize the MRE device, the schematic of which is shown in Figure 6. The generated force of the Dahl model is given as follows:

(33) $$F=c_{0}\dot{x}+k_{0}x+\alpha z$$

where *c*_0_ and *k*_0_ denote the viscous and stiffness coefficients, respectively; *α* denotes the scaling factor for the shape of hysteresis loops; *z* is the intermediate variable and its expression is show in Equation (34):

(34) $$\dot{z}=\rho \left(-\left|\dot{x}\right|z+\dot{x}\right)$$

where *ρ* is parameter related to the stiffness of the system. The Dahl model can be regarded as a particular case of the Bouc–Wen model. The modeling results in [26] indicated that the parameters *c*_0_, *k*_0_, and *α* are field dependent and can linearly change with the applied current *I*, which can be formulated as follows:

(35) $$k_{0}=k_{01}+k_{02}I$$

(36) $$c_{0}=c_{01}+c_{02}I$$

(37) $$\alpha =\alpha_{1}+\alpha_{2}I$$

### 2.7. LuGre Friction Model

**Figure 7:**
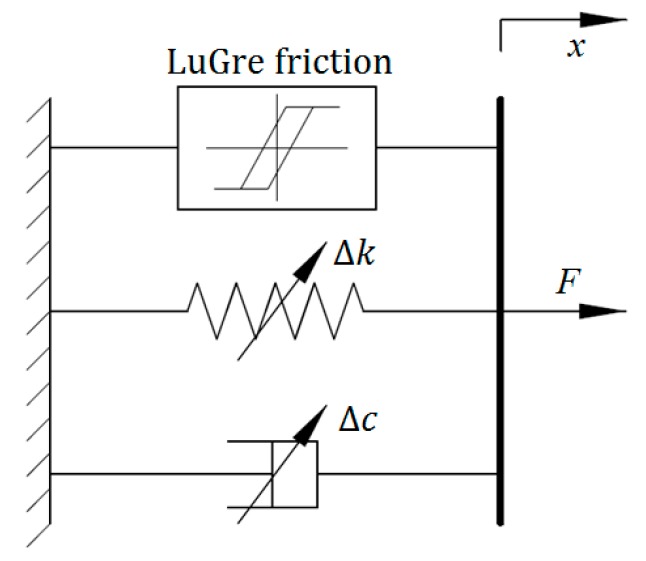
Improved LuGre friction model.

The LuGre friction model, as an extension of the Dahl model, was designed to characterize the friction dynamics of magnetorheological materials. In [33], it was employed to model the dynamic behavior of an MRE isolator. Figure 7 shows the configuration of the improved LuGre friction model for modeling of MRE devices. It can be seen that the model consists of a viscous dashpot, a linear spring element, and a LuGre friction component. The force generated from the model can be formulated as:

(38) $$F=\frac{\beta }{\alpha }y+c_{0}\dot{x}+k_{0}x+\frac{\varepsilon }{\alpha }\dot{y}$$

where *c*_0_ and *k*_0_ denote the viscous and stiffness coefficients, respectively. *α*, *β*, and *ε* are nondimensional parameters to regulate the size and shape of the hysteresis loops. *y* is an evolutionary variable that can be calculated via solving the following differential equation:

(39) $$\frac{1}{\alpha }\dot{y}=\dot{x}-\left|\dot{x}\right|y$$

Sensitivity analysis results in [33] indicated that parameters *k*_0_, *ε*, and *α* are more sensitive than other parameters in the improved LuGre friction model for MRE device with respect to external current. Accordingly, *k*_0_, *ε*, and *α* can be represented as the functions of the applied current, while other parameters are regarded as the constants due to minor effects on the model output. The related mathematical equations are given as follows:

(40) $$k_{0}=k_{0a}+k_{0b}I$$

(41) $$\varepsilon =\varepsilon_{0}+\varepsilon_{1}I+\varepsilon_{2}I^{2}$$

(42) $$\alpha =\alpha_{01}e^{-\alpha_{02}I}$$

### 2.8. Strain Stiffening Model

**Figure 8:**
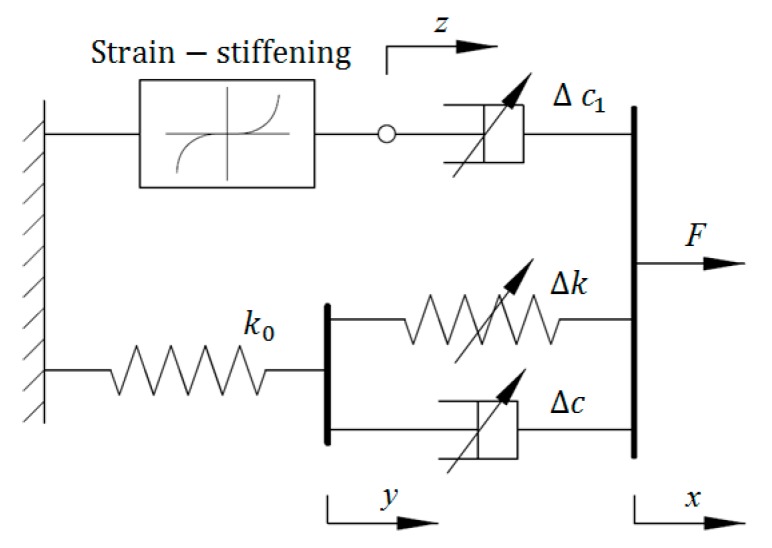
Strain stiffening model.

To avoid the complicated calculation in BW-based models due to a large number of identified parameters, Li and Li proposed a novel model to characterize the strain stiffening phenomenon in the force response of MRE isolator [22], in which the power law function is employed. As observed in Figure 8, this model can be separated into two parts. The upper part is the standard three-parameter solid element to model the viscoelastic characteristics of the MRE response, while the lower part is a dashpot connected to a power function element to illustrate the strain stiffening behavior of the device. The mathematical expressions of this model are shown in Equations (43)–(45).

(43) $$F=\alpha z^{3}+k_{1}y$$

(44) $$c_{0}\left(\dot{x}-\dot{y}\right)+k_{0}\left(x-y\right)=k_{1}y$$

(45) $$c_{1}\left(\dot{x}-\dot{z}\right)=\alpha z^{3}$$

where *k*_0_ and *k*_1_ denote the stiffness coefficients; *c*_0_ and *c*_1_ denote the viscous coefficients; *α* denotes the scaling factor for strain stiffening phenomenon of the response of MRE isolator. Two intermediate variables *y* and *z* can be obtained via calculating two nonlinear differential equations.

(46) $$\dot{y}=\frac{k_{0}}{c_{0}}x+\dot{x}-\frac{\left(k_{0}+k_{1}\right)}{c_{0}}y$$

(47) $$\dot{z}=\dot{x}-\frac{\alpha }{c_{1}}z^{3}$$

In this model, all the parameters are field-dependent, which can be expressed by first-order polynomial functions of applied current.

### 2.9. Hyperbolic Hysteresis Model

**Figure 9:**
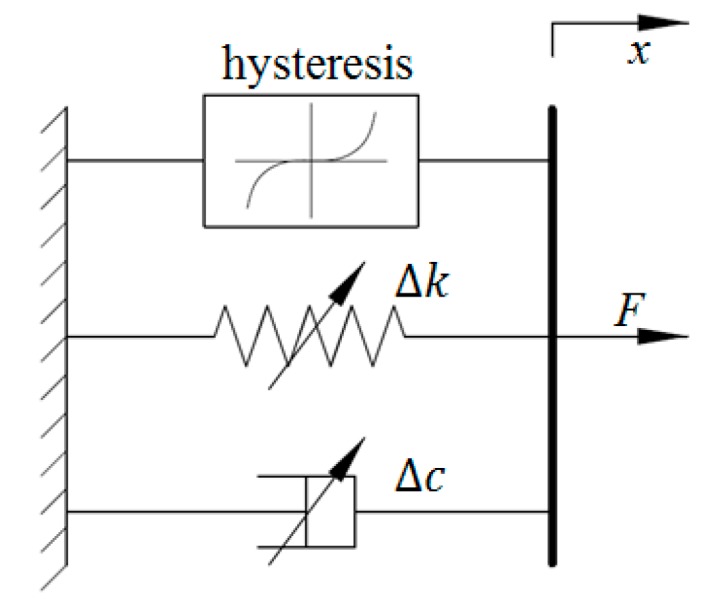
Hyperbolic hysteresis model.

To avoid a large amount of calculation in the BW, improved BW, Dahl, improved LuGre friction and strain stiffening models due to highly nonlinear differential equations in model expressions, Yu et al. developed a simple hyperbolic sine function-based hysteresis model for the nonlinear behavior of MRE device [30]. Figure 9 gives the structure of this model, in which a hyperbolic element is employed to replace the BW element in classical BW model. The main benefit of this model is to avoid calculating the differential equations, which may result in the iteration error. The related mathematical equations about the hyperbolic hysteresis model are provided as follows:

(48) $$F=k_{1}x+c_{1}\dot{x}+\alpha \sin h\left(\beta x\right)$$

where *k*_1_ and *c*_1_ denote the stiffness and viscous coefficients, respectively; *α* denotes the hysteresis scaling factor; *β* is a parameter related to the slope of the hysteresis loops; sinh denotes the hyperbolic sine operation. Similar to other phenomenological models, the parameters *k*_1_, *c*_1_, and *α* are field-dependent and their relationships with applied current *I* are shown in Equations (49)–(51).

(49) $$k_{1}=k_{11}+k_{12}I$$

(50) $$c_{1}=c_{11}+c_{12}I$$

(51) $$\alpha =\alpha_{1}+\alpha_{2}I$$

### 2.10. Model Summary

Table 1 summarizes the up-to-date phenomenological models for the MRE material and devices. Each model is described with advantages and/or disadvantages, which is dependent on the practical application.

## 3. Dynamic Tests of Magnetorheological Elastomer Device

### 3.1. Design of the MRE Isolator

**Figure 10:**
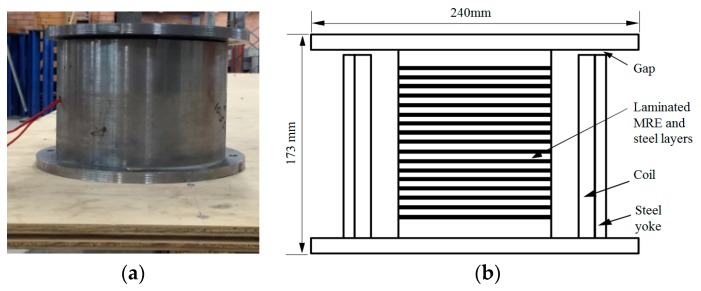
Design of the prototype MRE isolator. (**a**) Photo of the device; (**b**) Cross-section of the device.

Taking advantage of the field-dependent properties of the MREs, a prototype MRE device called MRE isolator was designed and fabricated [22]. As shown in Figure 10, the laminated core structure of the prototype device consists of 24 sheets of steel and 25 layers of MRE sheets. The MRE adopted for this device consists of silicon rubber (Selleys Pty. LTD, Padstow, New South Wales, Australia), silicon oil type 378,364 (Sigma-Aldrich Pty. LTD, L’ lsle D’Abeau Chesnes, St. Quentin Fallavier Cedex, France), and carbonyl iron particles type C3528 (Sigma-Aldrich Pty. LTD). The diameter of the iron particle is between 3 and 5 µm. The volume fraction of the iron particles is 22.9%. All the steel and MRE sheets are of 1 mm thickness with 120 mm diameter and bonded alternately. This structure allows flexible horizontal shear movements while maintaining a high vertical load capacity. Two thick endplates are placed at the two ends of the laminated core and serve as the connection between the top plate and bottom plate. Between the top plate and the yoke, a 5 mm gap is kept to eliminate the friction between the two steel parts. The coil is positioned inside of the yoke with 15 mm distance from the laminated core which allows the device to perform a shear test up to 60% shear strain. The 2900-turn winding of coil works as the source of magnetic field to generate sufficient and controllable MR effect. The maximum vertical load capacity is estimated as 50 kg at the weakest case which is 15 mm shear displacement and no current applied scenario.

### 3.2. Dynamic Test of MRE Device

**Figure 11:**
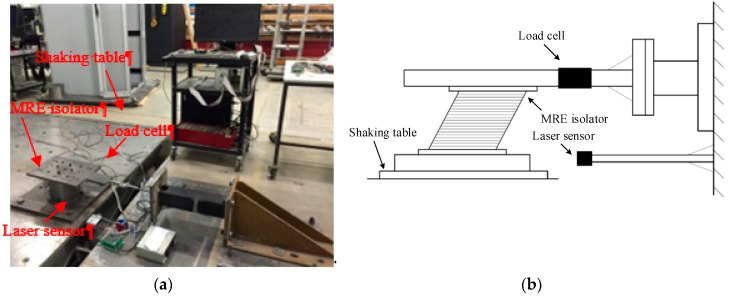
Experimental setup. (**a**) Setup photo; (**b**) Schematic of the setup.

The performance of the MRE isolator was evaluated and characterized by a series of experimental tests utilizing the experimental setup depicted in Figure 11. The isolator, either in dynamic or quasistatic mode, was applied with horizontal loadings that were provided by a shaking table, where the MRE isolator is mounted and moves along the same motion. The lateral load applied to the isolator is measured by a load cell that was installed to a fixed reaction rig. A laser sensor was installed parallel to the shaking table to measure the displacement response. In order to eliminate the undesired inertia force in the measurements, the top plate of the MRE isolator and the load cell remained motionless during the test process. A DC power supply with the capacity of 240 V and 5.3 A was utilized to provide DC current to energize the magnetic coil. During the testing, the current applied to the magnetic coil was adjusted by a slider and the current output from the slider was monitored by a multimeter.

The dynamic tests of the MRE isolator were conducted in the Structures Lab, University of Technology Sydney, based on displacement control. Because the main frequency range of earthquakes is below 5 Hz, the experimental tests were designed with excitation frequencies between 0.1 and 4 Hz. In this study, the harmonic signals with 0.1, 1, and 4 Hz frequencies were used to excite the device. The loading amplitudes are 2, 4, and 8 mm, corresponding to 8%, 16%, and 32% shear strain. For each input case, four magnetic fields that were generated by different currents (0–3 A) were applied to the device to test its performance. In order to ensure that the stable performance of the device is captured for each case, at least two cycles were measured for each single loading case. In order to ensure that the test results were accurate and consistent, each set of testing was repeated three times independently. Hence, there were 36 tests in total. The coil generates a great amount of heat due to its high resistance (about 43 Ω) in the current design. Generally, MRE materials are able to normally function under high temperature, since the matrix of MRE is usually silicon rubber that offers good resistance to temperatures up to 300 °C. However, the mechanical properties of the MRE, i.e., storage modulus, stiffness, and damping, will slightly decrease with the increase of temperature. Hence, the temperature should be maintained at a relatively constant level by temperature control for different sets of tests. In this work, after each set of test, 15–20 min break was taken for cooling the device. For the data acquisition, the sampling rate was set at 256 Hz to capture all test results, including the dynamic tests.

### 3.3. Test Results

**Figure 12:**
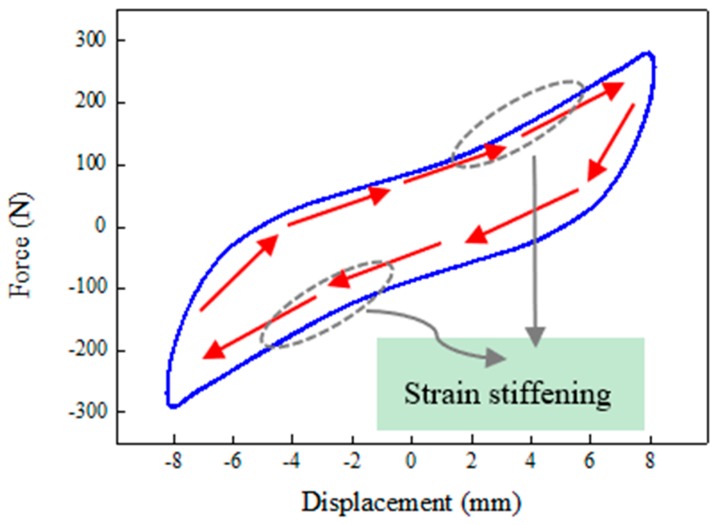
Dynamic responses of the MRE isolator under 4 Hz excitation frequency, 8 mm loading amplitude, and 2 A applied current. (**a**) Force–displacement loop, and (**b**) force–velocity loop.

Figure 12 shows an example of testing result, which demonstrates typical force–displacement/velocity responses of the MRE isolator when the excitation frequency is 4 Hz, the amplitude is 8 mm, and the applied current is 2 A. It is clearly observed that the device displays hysteretic loops in the shear force–displacement/velocity responses, which has the characteristic of strain stiffening under the influence of external current, as illustrated in previous studies. The interpretation of the phenomenon of strain stiffening is put down to the fact that the polymer chain has very constrained ductility for the natural rubber. Apart from the impedance from the polymeric matrix, the ferromagnetic particles are constrained by the interaction forces from neighboring ferromagnetic particles due to the magnetic field. This will lead to the reduced movement of the polymer chain. When the MRE is under low current conditions or in the absence of current, the phenomenon of strain stiffening is minimal and the MRE shows linear viscoelastic feature represented by a hysteretic ellipse loop. The amplitude and shape of the force–displacement/velocity loop are related to the loading amplitude and rate as well as the applied current level. Hence, the phenomenological models for the MRE device should be able to track the change of the hysteresis loop caused by loading condition. Based on the experimental data, the performances of nine models are evaluated and compared, the results of which will be specifically provided in Section 4.

## 4. Modeling Results and Discussions

**Figure 13:**
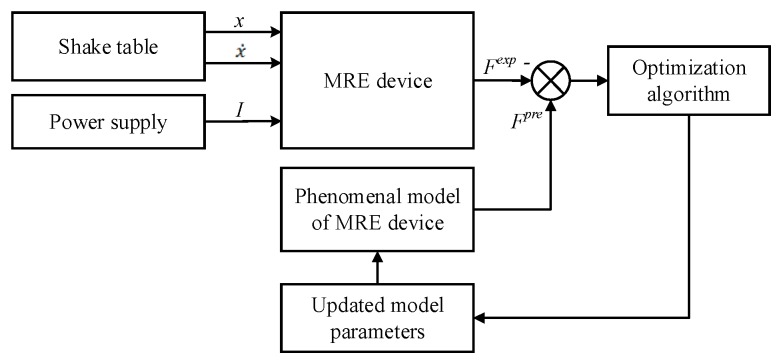
Schematic of model parameter identification.

The performances of the phenomenological models for MRE device can be evaluated using the experimental data with different loading scenarios collected in Section 3. The model parameters should be identified first to make the model predictions agree with experimental results. The strategy of the model parameter identification is to fit the force–displacement responses as close as possible to the measured force–displacement loops in the dynamic tests. The process of model parameter identification can be regarded as solving a multidimensional optimization problem, which is shown in Figure 13. The variables of the optimization problem are the parameters of phenomenological models for MRE device. Before the optimization problem is resolved, the fitness function should be determined since different fitness functions can lead to different convergences of the optimization solution. In this study, the root mean square error (RMSE) between measured force responses and model predictions in one loading cycle is employed as the fitness function of the optimization problem with the following expression:

(52) $$RMSE=\sqrt{\frac{1}{N_{f}}\overset{N_{f}}{\underset{k=1}{\sum }}{\left[F^{exp}\left(k\right)-F^{pre}\left(k\right)\right]}^{2}}$$

where *N_f_* denotes the sample number in one loading cycle; *F^exp^*(*k*) and *F^pre^*(*k*) denote *k*th measured force in the experiments and the force predicted from the model, respectively. Generally, the RMSE represents the differences of the second moment of differences between the model-predicted values and experimental observations. It can be seen from Equation (53) that the RMSE is always positive and the value of 0 indicates the best fit to the data. Accordingly, it can be used to identify the model parameters via comparing the predictive errors for a special dataset. Moreover, the improved Euler method is adopted to solve the differential equations in some phenomenological models for MRE device, such as the rheological, BW, improved BW, Dahl, improved LuGre friction, and strain stiffening models. The expressions for solving differential equations using the improved Euler method are provided as follows.

(53) $$y\left(k+1\right)=y\left(k\right)+\Delta t\cdot \dot{y}\left(k\right)+O\left(\Delta t^{2}\right)$$

(54) $$z\left(k+1\right)=z\left(k\right)+\Delta t\cdot \dot{z}\left(k\right)+O\left(\Delta t^{2}\right)$$

where ∆*t* denotes the sampling time interval.

**Figure 14:**
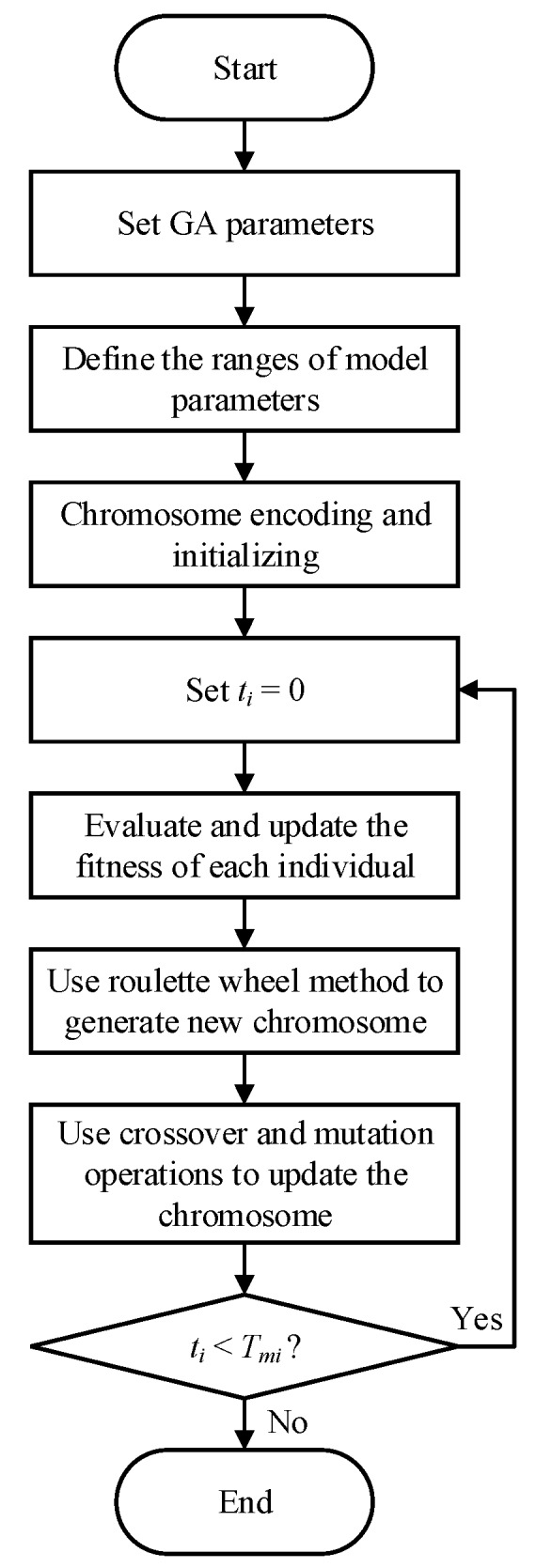
Flowchart of the genetic algorithm (GA) to identify model parameters.

To solve the optimization problems for optimal model parameters, evolutionary optimization algorithms can be considered because of independent requirements on derivative and analytic model expressions [39]. To date, several swarm-based algorithms have been successfully employed in model parameter optimization of MR fluids dampers, including particle swarm optimization (PSO) [40], genetic algorithm (GA) [41,42], fruit fly algorithm (FFA) [43], charged system search (CSS) [44], enriched imperialist competitive algorithm (EICA) [45], differential evolution (DE) algorithm [46], etc. However, solving the optimization problems for MRE devices are rarely reported. Zhu et al. adopted least square (LS) method to identify the magneto-viscoelastic model of MRE device [47]. Yang et al. utilized the GA to identify the Bouc–Wen model for MRE isolator [31]. Similarly, PSO and FFA have been applied in model identification of MRE devices [33,48]. Among existing methods, the GA is the most commonly used due to its benefits of easy coding and fast convergence [49,50]. Accordingly, in this study, it is selected to solve the optimization problem for model parameter identification of the MRE device. The procedure of using GA to identify model parameters of the MRE device is summarized as follows, and is shown in Figure 14.

Step 1. Determine the fitness function and model parameters to be identified.
Step 2. Set the algorithm parameters of GA: the number of chromosome *N_c_*= 30, mutation probability *p_m_* = 0.01, crossover probability *p_c_* = 0.7, maximum iteration number *T_mi_* = 200.
Step 3. Define the ranges of the parameters to be identified.
Step 4. Encode the chromosomes for the model parameters. Initialize the chromosomes randomly and the initial iteration value is set as *t_i_* = 0.
Step 5. Evaluate the fitness value of each individual and compare the current value with previous one.
Step 6. Use the roulette wheel method to choose part of the chromosome to generate new chromosome.
Step 7. Conduct the crossover and mutation operations to update the chromosome.
Step 8. Check the stopping criterion. If the current iteration number is smaller than the maximum iteration number, go back to Step 5; otherwise, the algorithm is terminated.

**Figure 15:**
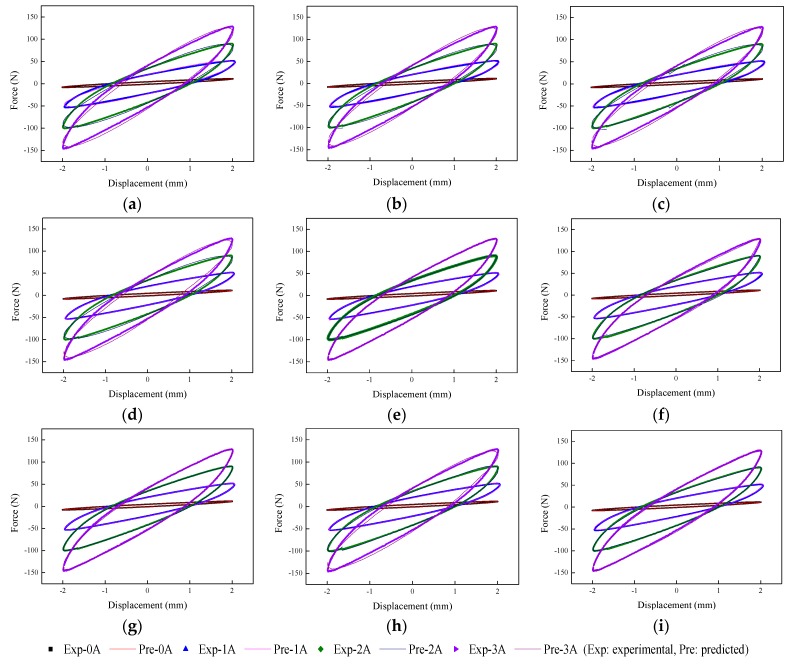
Comparisons of force–displacement responses between experimental results and model predictions when the loading frequency and amplitude are 0.1 Hz and 2 mm. (**a**) Kelvin–Voigt model, (**b**) four-parameter viscoelastic model, (**c**) rheological model, (**d**) BW model, (**e**) improved BW model, (**f**) Dahl model, (**g**) improved LuGre friction model, (**h**) strain stiffening model, and (**i**) hyperbolic hysteresis model.

**Figure 16:**
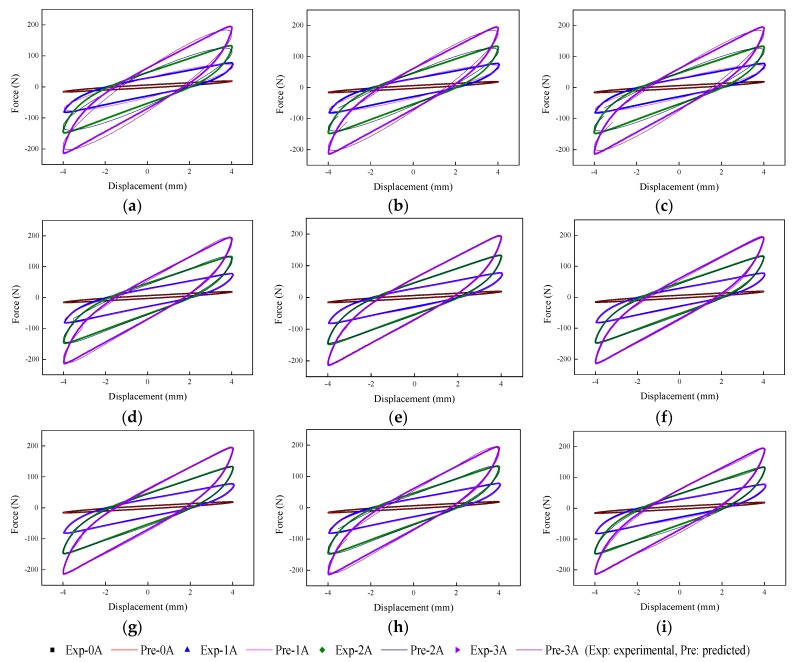
Comparisons of force–displacement responses between experimental results and model predictions when the loading frequency and amplitude are 0.1 Hz and 4 mm. (**a**) Kelvin–Voigt model, (**b**) four-parameter viscoelastic model, (**c**) rheological model, (**d**) BW model, (**e**) improved BW model, (**f**) Dahl model, (**g**) improved LuGre friction model, (**h**) strain stiffening model, and (**i**) hyperbolic hysteresis model.

**Figure 17:**
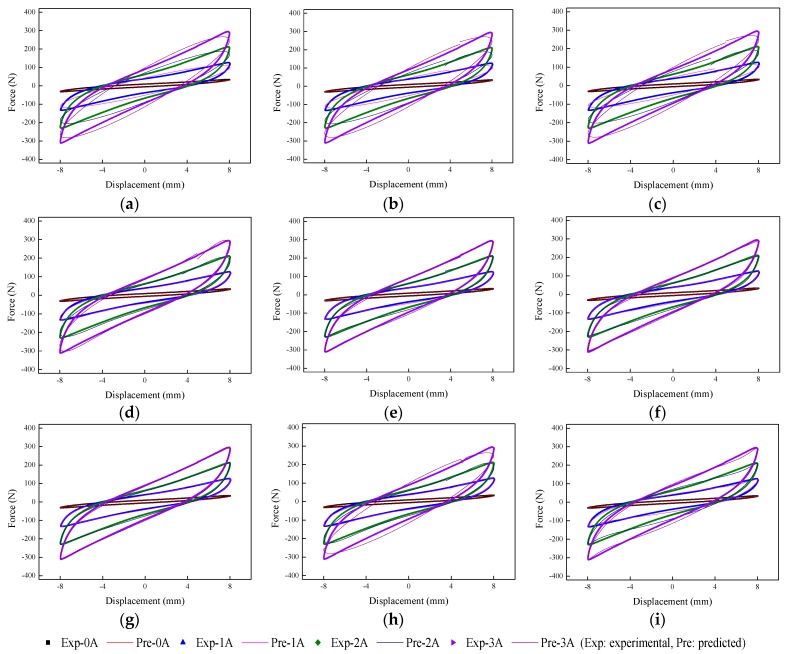
Comparisons of force–displacement responses between experimental results and model predictions when the loading frequency and amplitude are 0.1 Hz and 8 mm. (**a**) Kelvin–Voigt model, (**b**) four-parameter viscoelastic model, (**c**) rheological model, (**d**) BW model, (**e**) improved BW model, (**f**) Dahl model, (**g**) improved LuGre friction model, (**h**) strain stiffening model, and (**i**) hyperbolic hysteresis model.

**Figure 18:**
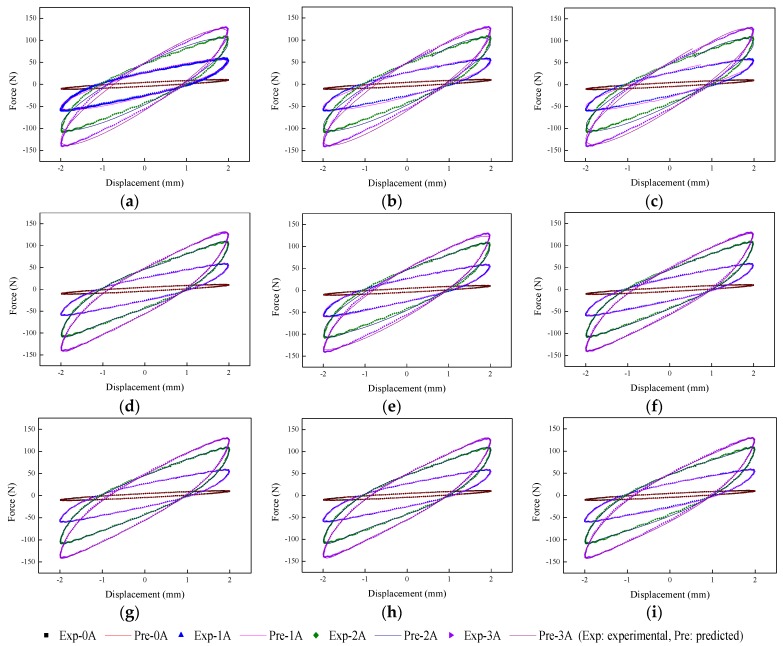
Comparisons of force–displacement responses between experimental results and model predictions when the loading frequency and amplitude are 1 Hz and 2 mm. (**a**) Kelvin–Voigt model, (**b**) four-parameter viscoelastic model, (**c**) rheological model, (**d**) BW model, (**e**) improved BW model, (**f**) Dahl model, (**g**) improved LuGre friction model, (**h**) strain stiffening model, and (**i**) hyperbolic hysteresis model.

**Figure 19:**
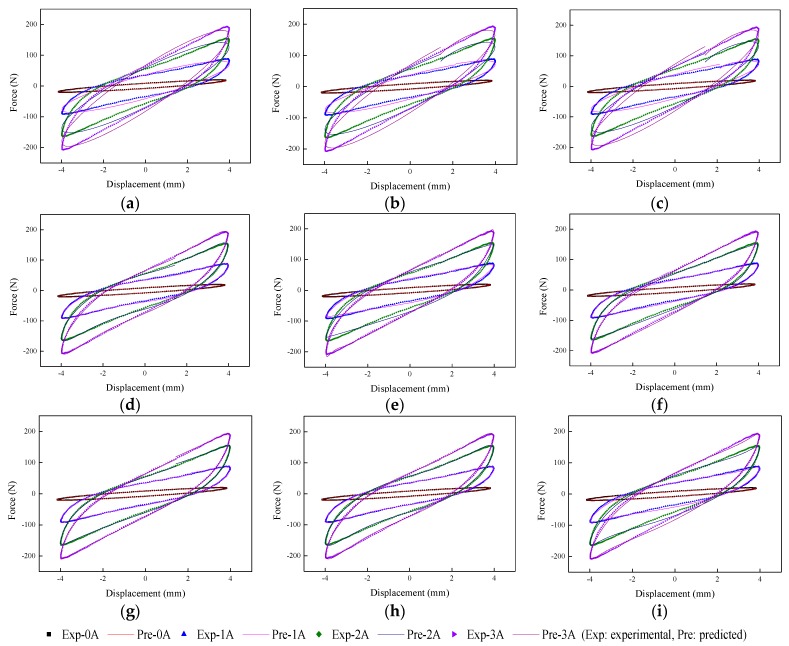
Comparisons of force–displacement responses between experimental results and model predictions when the loading frequency and amplitude are 1 Hz and 4 mm. (**a**) Kelvin–Voigt model, (**b**) four-parameter viscoelastic model, (**c**) rheological model, (**d**) BW model, (**e**) improved BW model, (**f**) Dahl model, (**g**) improved LuGre friction model, (**h**) strain stiffening model, and (**i**) hyperbolic hysteresis model.

**Figure 20:**
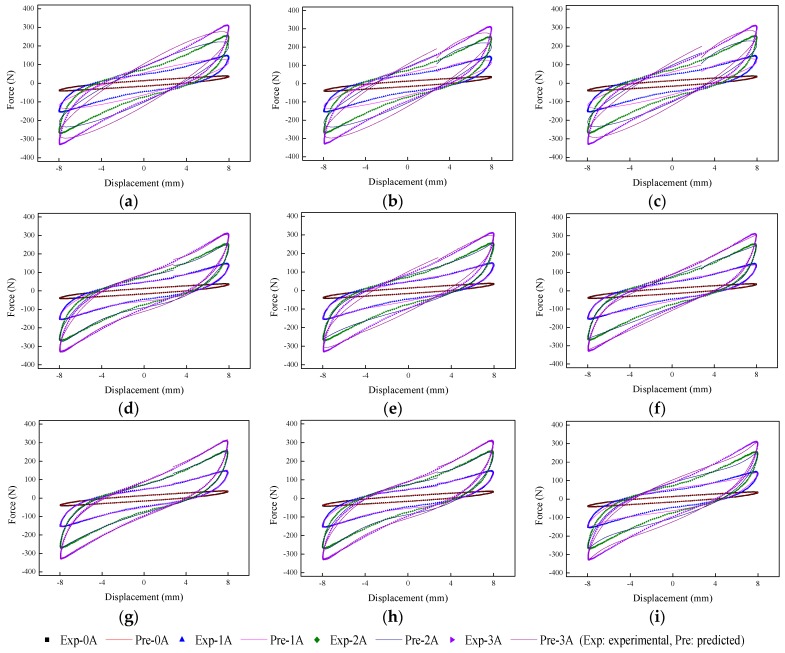
Comparisons of force–displacement responses between experimental results and model predictions when the loading frequency and amplitude are 1 Hz and 8 mm. (**a**) Kelvin–Voigt model, (**b**) four-parameter viscoelastic model, (**c**) rheological model, (**d**) BW model, (**e**) improved BW model, (**f**) Dahl model, (**g**) improved LuGre friction model, (**h**) strain stiffening model, and (**i**) hyperbolic hysteresis model.

**Figure 21:**
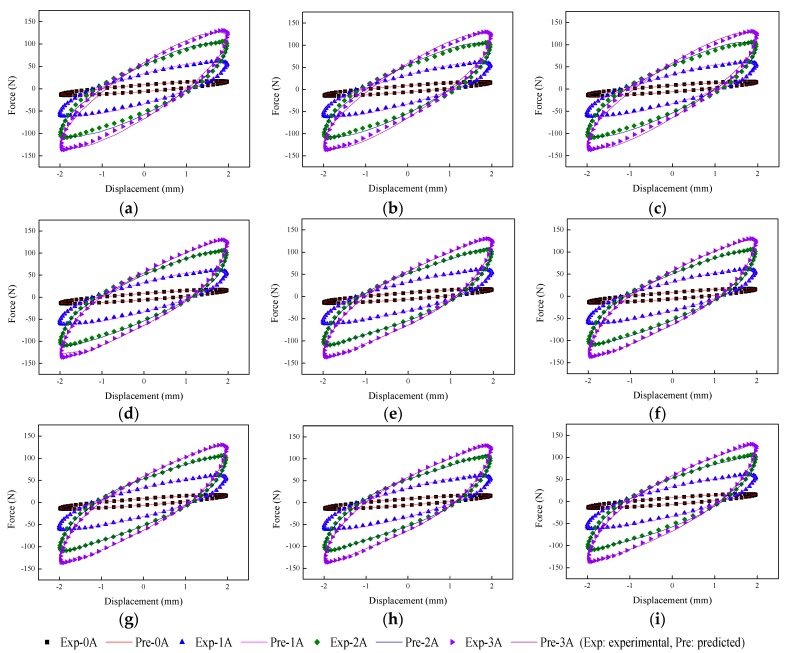
Comparisons of force–displacement responses between experimental results and model predictions when the loading amplitude is 2 mm. (**a**) Kelvin–Voigt model, (**b**) four-parameter viscoelastic model, (**c**) rheological model, (**d**) BW model, (**e**) improved BW model, (**f**) Dahl model, (**g**) improved LuGre friction model, (**h**) strain stiffening model, and (**i**) hyperbolic hysteresis model.

**Figure 22:**
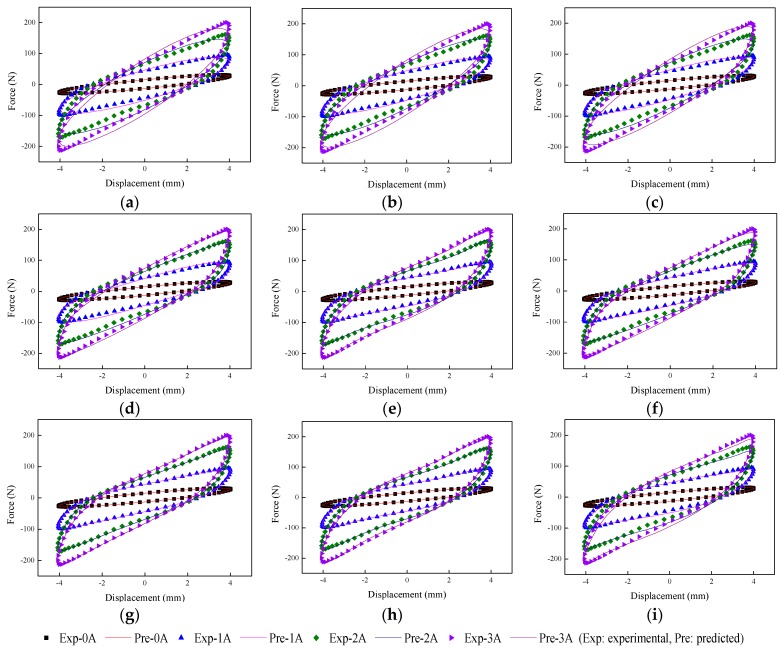
Comparisons of force–displacement responses between experimental results and model predictions when the loading amplitude is 4 mm. (**a**) Kelvin–Voigt model, (**b**) four-parameter viscoelastic model, (**c**) rheological model, (**d**) BW model, (**e**) improved BW model, (**f**) Dahl model, (**g**) improved LuGre friction model, (**h**) strain stiffening model, and (**i**) hyperbolic hysteresis model.

**Figure 23:**
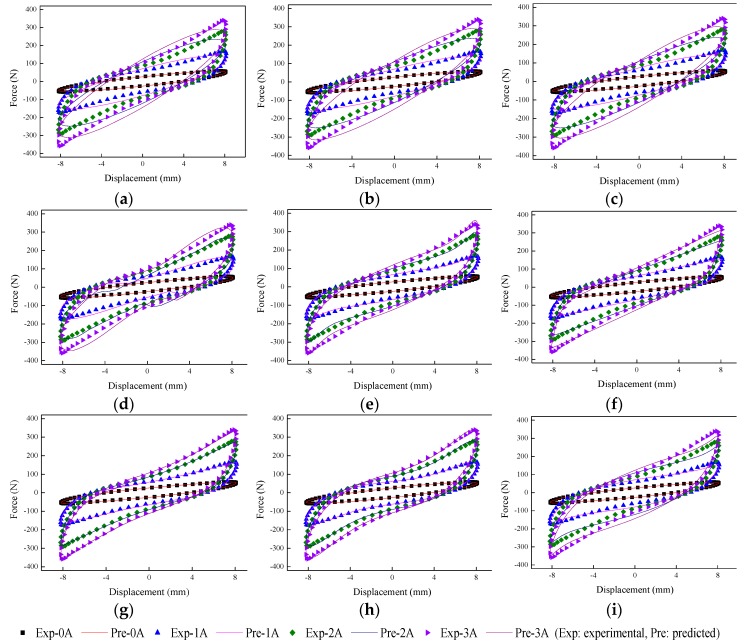
Comparisons of force–displacement responses between experimental results and model predictions when the loading amplitude is 8 mm. (**a**) Kelvin–Voigt model, (**b**) four-parameter viscoelastic model, (**c**) rheological model, (**d**) BW model, (**e**) improved BW model, (**f**) Dahl model, (**g**) improved LuGre friction model, (**h**) strain stiffening model, and (**i**) hyperbolic hysteresis model.

The implementation of the GA is based on Matlab v2012b. The data tested from the MRE isolator with various loading conditions are sent to nine phenomenological models for parameter identification. The modeling results are shown in Figure 15, Figure 16, Figure 17, Figure 18, Figure 19, Figure 20, Figure 21, Figure 22 and Figure 23, where the force–displacement loops with different loading frequencies and amplitudes as well as different applied currents are compared between measured and predicted results. In the figures, the straight lines denote the model prediction results, while discrete points denote the experimentally measured forces. It is noticeable that all the models exhibit high accuracy in characterizing force–displacement responses under low loading amplitude (2 mm). Especially, in the absence of applied current (0 A) or under small current supply (1 A), the predicted forces match the measured results very well and all the models perfectly portray the field-dependent characteristic of the MRE device. However, when the loading amplitude is increased to 4 mm and 8 mm, the Kelvin–Voigt model, four-parameter viscoelastic model, and rheological model are not capable of effectively tracking the shear forces of the device under high current supply (2 and 3 A), compared to other models. The main reason leading to this phenomenon is that these three models can only effectively characterize the linear viscoelastic behavior of the MRE device when it is under small deformation (2 mm) without current supply. When the MRE device is under large deformation and high current supply, it presents the unique behavior of strain stiffening, which cannot be well characterized by the Kelvin–Voigt model, four-parameter viscoelastic model, or rheological model.

Effective stiffness (ES) and equivalent damping (ED) are two main parameters to evaluate the stiffness and energy dissipation properties of the MRE device. Accordingly, the comparisons of both indices under different loading conditions between experimental results and model predictions are conducted to assess the abilities of the phenomenological models to portray the field-dependent properties. Both ES and ED can be calculated based on the force–displacement loops with the following equations:

(55) $$ES=\frac{F_{max}-F_{min}}{x_{max}-x_{min}}$$

(56) $$ED=\frac{E_{d}}{2\pi^{2}A^{2}w}$$

where *x_max_* and *x_min_* denotes the maximum and minimum displacements, respectively. *F_max_* and *F_min_* denote the forces corresponding to *x_max_* and *x_min_*, respectively. *E_d_* denotes the enclosed area of the force–displacement loop, which indicates the energy dissipation in one cycle. *A* and *w* denote the excitation amplitude and frequency, respectively.

**Figure 24:**
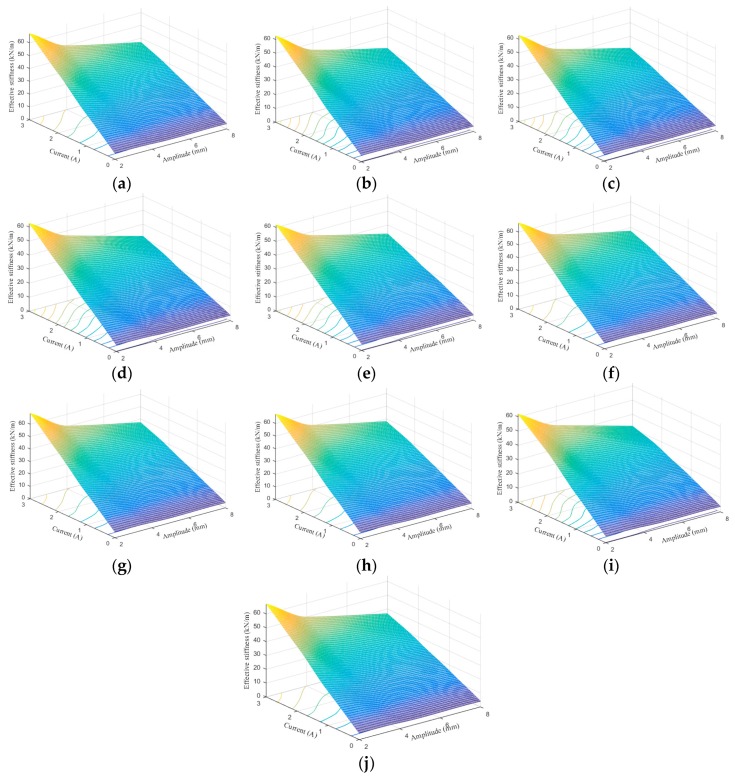
Effective stiffness estimation results of 0.1 Hz loading frequency. (**a**) Experimental measurement, (**b**) Kelvin–Voigt model, (**c**) four-parameter viscoelastic model, (**d**) rheological model, (**e**) BW model, (**f**) improved BW model, (**g**) Dahl model, (**h**) improved LuGre friction model, (**i**) strain stiffening model, and (**j**) hyperbolic hysteresis model.

**Figure 25:**
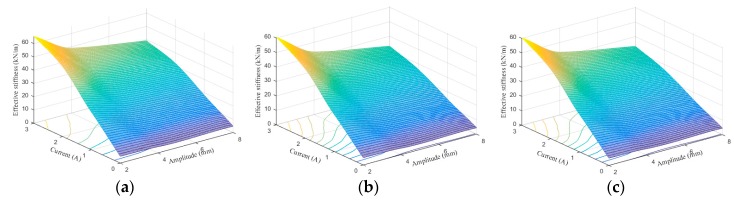

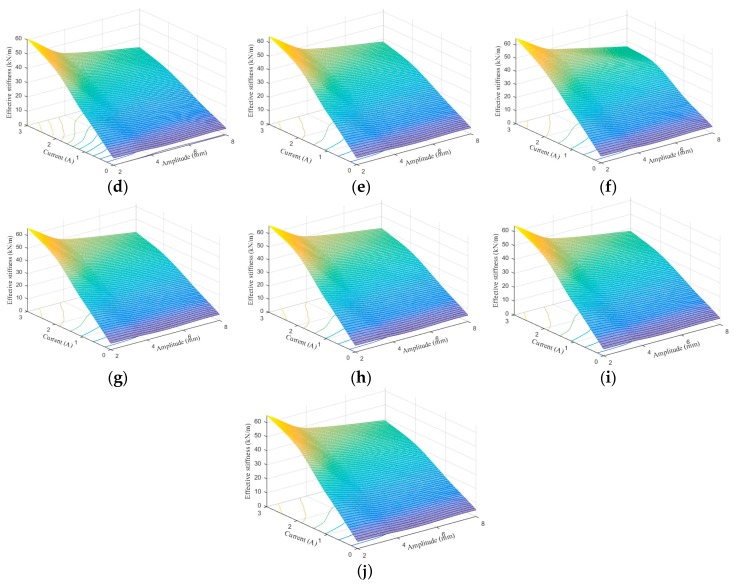
Effective stiffness estimation results of 1 Hz loading frequency. (**a**) Experimental measurement, (**b**) Kelvin–Voigt model, (**c**) four-parameter viscoelastic model, (**d**) rheological model, (**e**) BW model, (**f**) improved BW model, (**g**) Dahl model, (**h**) improved LuGre friction model, (**i**) strain stiffening model, and (**j**) hyperbolic hysteresis model.

**Figure 26:**
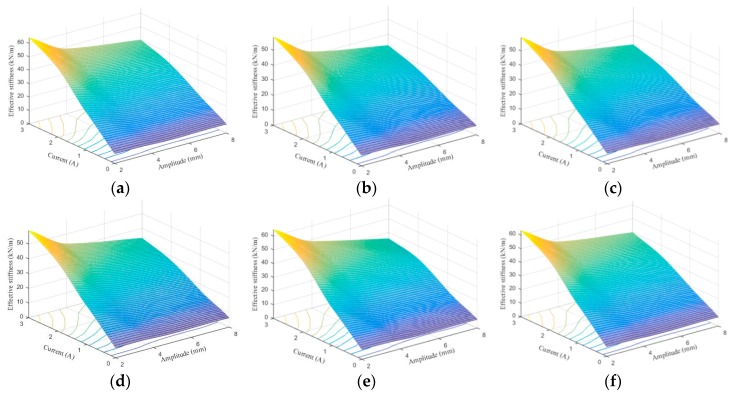

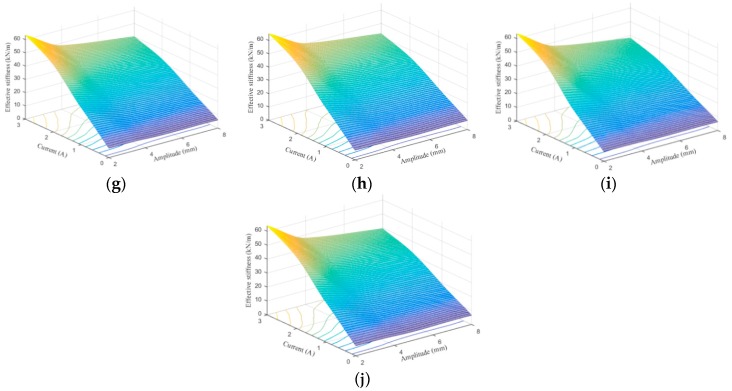
Effective stiffness estimation results of 4 Hz loading frequency. (**a**) Experimental measurement, (**b**) Kelvin–Voigt model, (**c**) four-parameter viscoelastic model, (**d**) rheological model, (**e**) BW model, (**f**) improved BW model, (**g**) Dahl model, (**h**) improved LuGre friction model, (**i**) strain stiffening model, and (**j**) hyperbolic hysteresis model.

**Figure 27:**
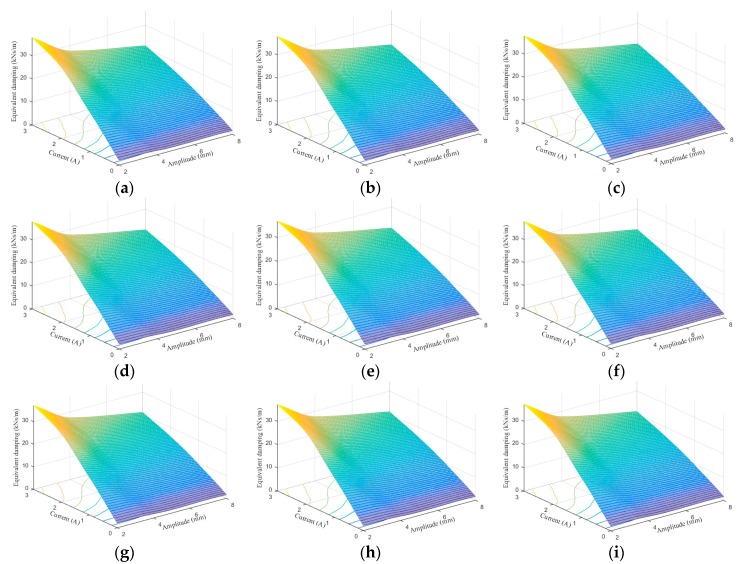

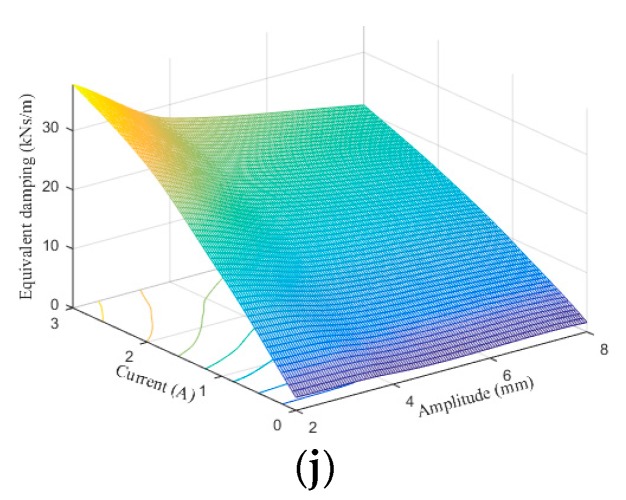
Equivalent damping estimation results of 0.1 Hz loading frequency. (**a**) Experimental measurement, (**b**) Kelvin–Voigt model, (**c**) four-parameter viscoelastic model, (**d**) rheological model, (**e**) BW model, (**f**) improved BW model, (**g**) Dahl model, (**h**) improved LuGre friction model, (**i**) strain stiffening model, and (**j**) hyperbolic hysteresis model.

**Figure 28:**
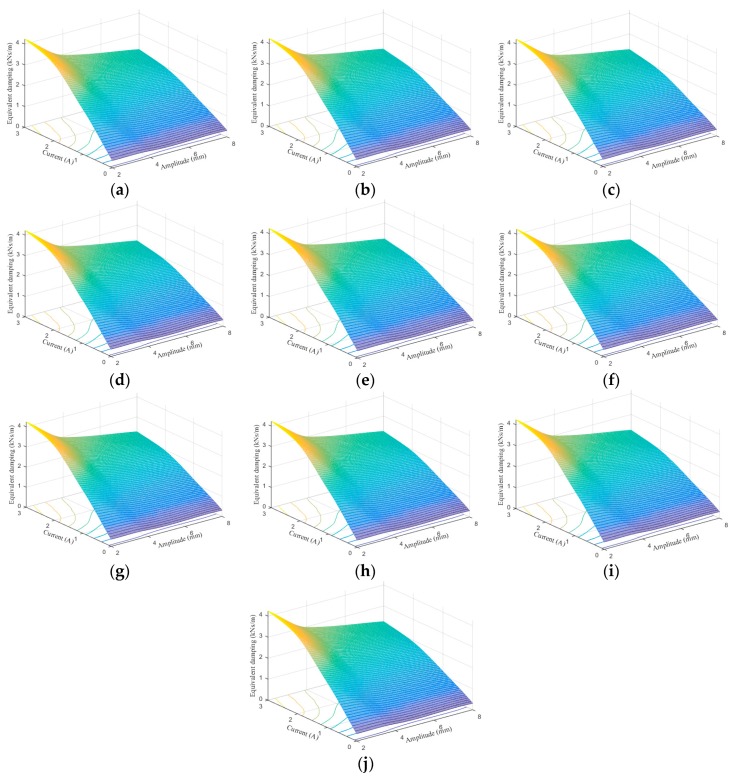
Equivalent damping estimation results of 1 Hz loading frequency. (**a**) Experimental measurement, (**b**) Kelvin–Voigt model, (**c**) four-parameter viscoelastic model, (**d**) rheological model, (**e**) BW model, (**f**) improved BW model, (**g**) Dahl model, (**h**) improved LuGre friction model, (**i**) strain stiffening model, and (**j**) hyperbolic hysteresis model.

**Figure 29:**
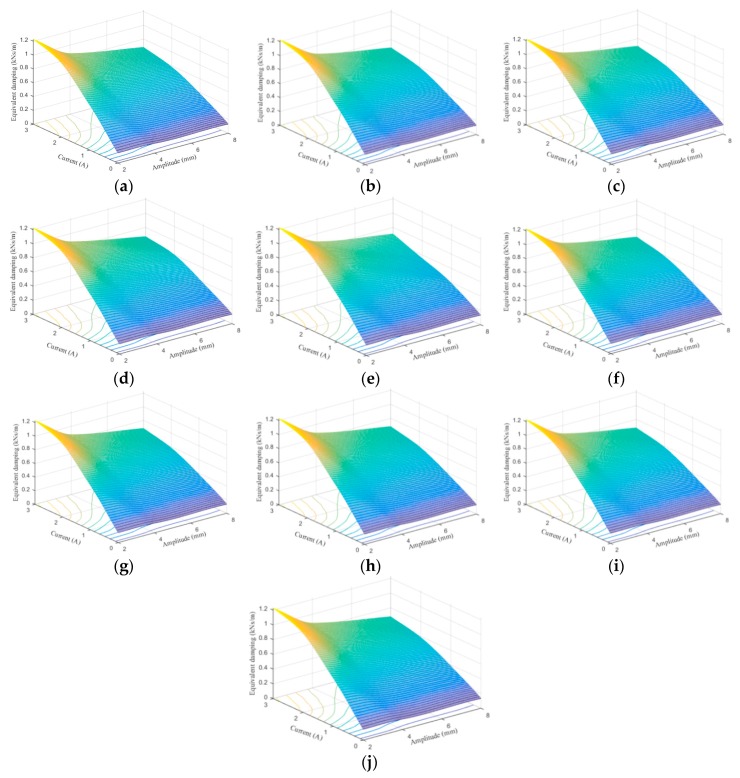
Equivalent damping estimation results of 4 Hz loading frequency. (**a**) Experimental measurement, (**b**) Kelvin–Voigt model, (**c**) four-parameter viscoelastic model, (**d**) rheological model, (**e**) BW model, (**f**) improved BW model, (**g**) Dahl model, (**h**) improved LuGre friction model, (**i**) strain stiffening model, and (**j**) hyperbolic hysteresis model.

Figure 24, Figure 25, Figure 26, Figure 27, Figure 28 and Figure 29 show the 3D plots of ES and ED of experimental tests and nine phenomenological models with different excitation frequencies and amplitudes as well as different applied current levels via cubic spline interpolation. It can be observed from the figures that with the increase of the excitation amplitude, the values of ES and ED undergo a slight deterioration, which is the Payne phenomenon [51]. This phenomenon is more obvious when the MRE device is supplied with high current levels. Different from the excitation amplitude, increasing current can contribute to significant improvements of ES and ED. From the comparisons between experimental and numerical results in terms of ES and ED, it can be concluded that all the phenomenological models are capable of effectively characterizing the change tendencies of ES and ED with loading amplitude and applied current level. However, for the quantitative evaluation of model performance, different phenomenological models have different modeling accuracies. For instance, the peak ES and ED should occur at the loading case of 4 Hz frequency, 2 mm amplitude, and 3 A current level, and the corresponding values are 64.1992 kN/m and 1.2277 kNs/m, respectively. The relative errors between experimental results and numerical results from nine phenomenological models are −8.44%, −7.76%, −7.69%, 1.04%, −1.34%, −1.23%, 1.50%, −0.80% and −0.30% for ES and −0.13%, 0.48%, −0.34%, 0.07%, −0.01%, 0.02%, −0.01%, −0.02%, and 0.08% for ED, respectively. Similar accuracy results can also be found in other loading cases. Apparently, the BW, improved BW, Dahl, improved LuGre friction, strain stiffening, and hyperbolic hysteresis models outperform the Kelvin–Voigt model, four-parameter viscoelastic model, and rheological model in predicting the ES and ED properties of the MRE device.

To further quantify the performance of the phenomenological models to predict the shear force of the MRE isolator under all the loading scenarios, regression analysis is conducted to evaluate the matching degree between experimental and numerical results. Here, the coefficient of determination (*R*^2^) index is utilized to assess the goodness of fit of the phenomenological model and its mathematical expression is given as follows:

(57) $$R^{2}=1-\frac{SAES}{TSS}$$

(58) $$SAES=\overset{N_{t}}{\underset{k=1}{\sum }}{\left[F^{exp}\left(k\right)-F^{pre}\left(k\right)\right]}^{2}$$

(59) $$TSS=\overset{N_{t}}{\underset{k=1}{\sum }}{\left[F^{exp}\left(k\right)-\frac{1}{N_{t}}\overset{N_{t}}{\underset{j=1}{\sum }}F^{exp}\left(j\right)\right]}^{2}$$

where *SAES* denotes the summation of absolute error squares, and *TSS* denotes the total summation of squares. Generally, the value of *R*^2^ should be in the range of [0, 1], where “0” means that the evaluated model has not shown the ability to demonstrate the variable to the response data, and “1” denotes the evaluate model has strong capacity to explain the variable to the response data. In practice, if the value of *R*^2^ is above 0.9, it indicates a good relationship between real and predicted data.

**Figure 30:**
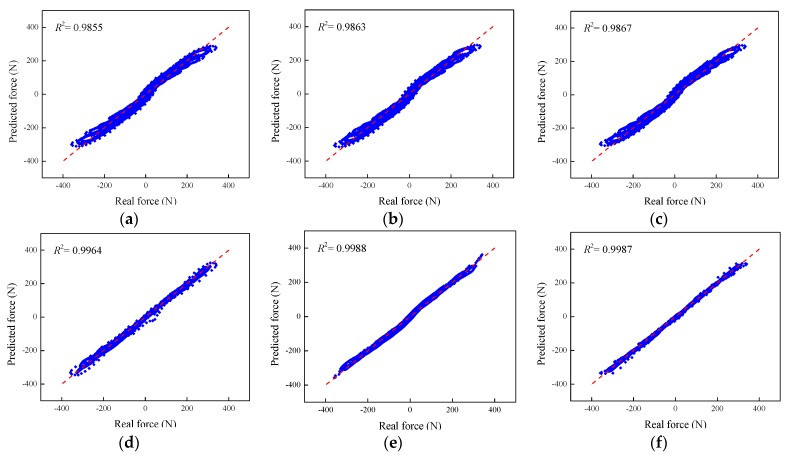

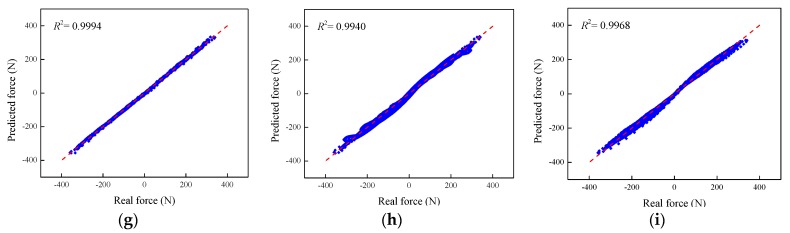
Regression analysis of the phenomenological models. (**a**) Kelvin–Voigt model, (**b**) four-parameter viscoelastic model, (**c**) rheological model, (**d**) BW model, (**e**) improved BW model, (**f**) Dahl model, (**g**) improved LuGre friction model, (**h**) strain stiffening model, and (**i**) hyperbolic hysteresis model.

Figure 30 shows the regression analysis results of the phenomenological models to predict the force of the MRE device with all the loading conditions. It is apparently observed from the figures that all the data points are evenly distributed on both sides of the regression line, which indicates the perfect matching degree between experimental measurements and predicted forces from phenomenological models. In regard to the coefficient of determination, the corresponding values of all the models are above 0.97. Accordingly, the numerical results from all the phenomenological models satisfactorily accord with the experimental results. Particularly for the improved BW, Dahl, improved LuGre friction, strain stiffening, and hyperbolic hysteresis models, their R-squared values are above 0.99, indicating high modeling accuracy.

**Figure 31:**
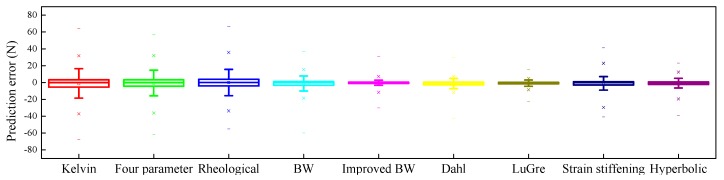
Prediction error distribution.

Figure 31 provides the statistical distributions of the absolute errors between the measured forces and numerical results for all the loading cases. From the figure, it is clearly observed that all the models have similar median values of absolute error, which approximate to zero. The improved LuGre friction model has the shortest range between the lower and upper boundaries, followed by the improved BW model and the Dahl model. Compared with other models, the Kelvin–Voigt model, four-parameter viscoelastic model, and rheological model have obvious abnormal points of absolute error, the values of which are above 40 N or below −40 N. Hence, their model accuracies are worse than those of other models. Among all the phenomenological models, the improved LuGre friction model performs the best in terms of prediction absolute error.

To comprehensively appraise the prediction capacities of the phenomenological models for characterizing the dynamic response of MRE device, apart from the R-square, more statistical indices should be considered, including RMSE, mean absolute percentage error (MAPE), and mean absolute error (MAE). The definition and mathematical expression of RMSE have been provided in Equation (52). MAPE is defined as the averaged absolute error of the predictions, while the MAE uses the standard deviation between the experimental result and numerical prediction. The calculation equations of the MAPE and MAE are shown in Equations (60) and (61):

(60) $$MAPE=\frac{1}{N_{t}}\overset{N_{t}}{\underset{j=1}{\sum }}\left|\frac{F^{exp}\left(j\right)-F^{pre}\left(j\right)}{F^{exp}\left(j\right)}\right|\times 100$$

(61) $$MAE=\frac{1}{N_{t}}\overset{N_{t}}{\underset{j=1}{\sum }}\left|F^{exp}\left(j\right)-F^{pre}\left(j\right)\right|$$

**Figure 32:**
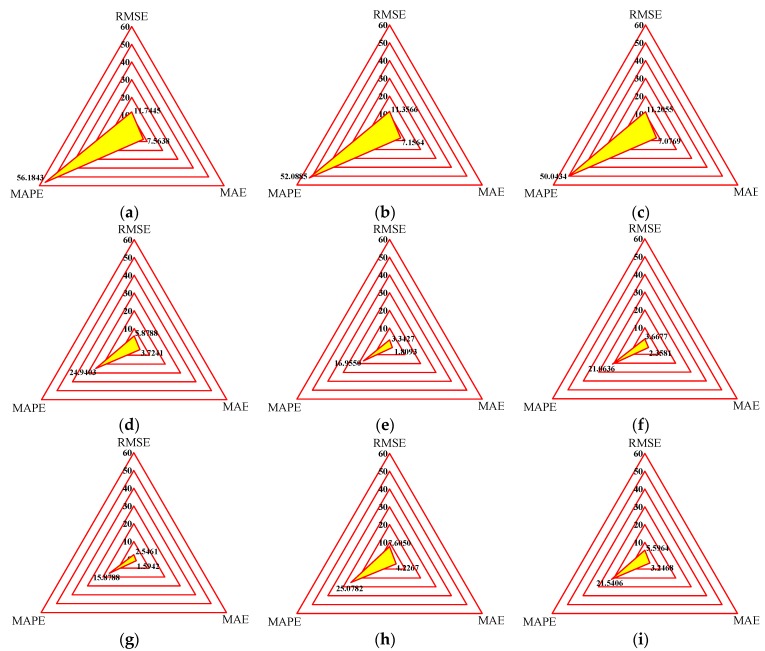
Statistical evaluation indices of phenomenological models. (**a**) Kelvin–Voigt model, (**b**) four-parameter viscoelastic model, (**c**) rheological model, (**d**) BW model, (**e**) improved BW model, (**f**) Dahl model, (**g**) improved LuGre friction model, (**h**) strain stiffening model, (**i**) hyperbolic hysteresis model.

Generally, the smaller the values of RMSE, MAPE, and MAE, the better the prediction ability of the model. Figure 32 shows the radar plots of three evaluation indices of different phenomenological models under all the loading cases. The enclosed area in yellow can be deemed as a comprehensive indicator to demonstrate the prediction accuracy of the model. Conspicuously, the improved LuGre friction model has the minimum enclosed area compared to other models. The corresponding RMSE, MAPE, and MAE of the improved LuGre friction model are 2.5461, 15.8788, and 1.5942, respectively. The relative deviations between the improved LuGre friction model and the Kelvin–Voigt model, four-parameter viscoelastic model, rheological model, BW model, improved BW model, Dahl model, improved LuGre friction model, strain stiffening model, and hyperbolic hysteresis model are 361.27%, 346.04%, 340.10%, 130.89%, 31.29%, 44.05%, 198.69%, and 119.80% for RMSE index, 253.83%, 228.04%, 215.16%, 57.07%, 6.78%, 32.65%, 57.94%, and 35.66% for MAPE index, and 374.46%, 348.90%, 343.92%, 133.60%, 13.49%, 47.92%, 165.13%, and 103.66% for MAE index, respectively. As a consequence, it can be concluded that the improved LuGre friction model outperforms other models and has the highest accuracy in modeling the dynamic behavior of the MRE device.

## 5. Conclusions

MRE is a novel type of smart material with real-time controllable damping and stiffness properties. This paper aims to evaluate different models for characterizing the nonlinear and hysteretic dynamics of the MRE device, which is of great importance to the design of a real-time controller. First of all, an in-depth literature review on up-to-date phenomenological models for characterizing MRE materials or associated devices is conducted. Several commonly used models are selected for performance investigation in prediction of the dynamic responses of an MRE device subjected to sinusoidal excitation loading. In addition, the hysteresis behavior of a prototype MRE isolator was experimentally explored using a shaking table. To fully evaluate this MRE device, several groups of dynamic tests were carried out with different excitation frequencies and amplitudes as well as different applied current levels. Then, the measured experimental data were used for the model identification and validation. The model parameters were identified using GA via minimizing the RMSE between experimental results and model predictions. The performances of the phenomenological models were assessed in terms of force–displacement hysteresis loops, effectiveness stiffness, and equivalent damping properties, as well as their statistical evaluation indices, such as R-squared, RMSE, MAE, and MAPE. Relevant conclusions are provided as follows:

(1) The results indicate that all the models show high accuracy in characterizing force–displacement responses under low excitation amplitude. However, the Kelvin–Voigt model, four-parameter viscoelastic model, and rheological model could not effectively track the strain hardening phenomenon of the MRE device under high current levels and large deformation.
(2) All the models are able to perfectly predict the variation tendencies of effective stiffness and equivalent damping properties of the MRE device with loading amplitude and applied current level, although the prediction accuracies have some variations between the different models.
(3) The improved LuGre friction model, improved BW model, and Dahl model have the best performances in terms of their absolute errors between experimental results and model predictions, with the shortest range between lower and upper boundaries.
(4) Based on the statistical evaluation indices, the improved LuGre friction model has the optimal performance with values of 0.9994 for R-squared, 2.5461 for RMSE, 1.5942 for MAE, and 15.8788 for MAPE.

## Figures and Tables

**Table 1:** Summary of phenomenological models of MRE materials/devices.

Model	Advantages and/or Disadvantages	Reference
Kelvin–Voigt model	The Kelvin–Voigt model is capable of effectively illustrating viscoelastic behavior of MRE system under given loading conditions. The drawback is that it lacks the ability to numerically characterize the material system.	[21]
Four-parameter viscoelastic model	The field-dependent modulus and damping capacities can be well characterized.	[28]
Rheological model	Besides the viscoelastic behavior, the field-dependent mechanical properties and interfacial slippage between polymer matrix and the particles are also considered in the rheological model.	[29]
Bouc–Wen model	The hysteretic behavior of MRE device can be well characterized. However, a large number of parameters should be identified and a highly nonlinear differential equation is incorporated that increases the challenge for the controller design.	[31]
Improved Bouc–Wen model	Compared to BW model, improved BW model introduces more parameters to better portray the hysteretic responses of MRE device.	[32]
Dahl model	The Dahl model can be regarded as a special case of the general BW model, and it has fewer model parameters than the BW model, with the benefit of good computational efficiency.	[26]
LuGre friction model	The friction dynamics of the MRE device can be modeled by LuGre friction model. Its main disadvantage is that the model lacks a term to describe the Stribeck effect [39], which may be apparent at low velocity areas.	[33]
Strain stiffening model	The strain stiffening phenomenon of MRE materials and devices can be well captured by this model. However, two differential equations in the model expression increase the challenge for model parameter identification.	[22]
Hyperbolic hysteresis model	The hyperbolic hysteresis model has simple model structure with only four parameters. The main advantage of this model is that it does not have any differential equation in the model.	[30]

## Abbreviations

MRE	Magnetorheological elastomer
AI	Artificial intelligence
BW	Bouc–Wen
VSDI	Variable stiffness and damping isolator
GA	Genetic algorithm
ES	Effective stiffness
ED	Equivalent damping
RMSE	Root mean square error
SAES	Summation of absolute error squares
TSS	Total summation of squares
MAPE	Mean absolute percentage error
MAE	Mean absolute error
